# Erythromycin-nonsusceptible *Streptococcus pneumoniae* in Children, 1999–2001

**DOI:** 10.3201/eid1106.050119

**Published:** 2005-06

**Authors:** M. Catherine McEllistrem, Jennifer M. Adams, Kathleen Shutt, Laurie T. Sanza, Richard R. Facklam, Cynthia G. Whitney, James H. Jorgensen, Lee H. Harrison

**Affiliations:** * University of Pittsburgh Graduate School of Public Health and School of Medicine, Pittsburgh, Pennsylvania, USA;; † Johns Hopkins University Bloomberg School of Public Health, Baltimore, Maryland, USA;; ‡ Centers for Disease Control and Prevention, Atlanta, Georgia, USA;; § University of Texas Health Science Center, San Antonio, Texas, USA

**Keywords:** molecular epidemiology, bacteremia, Antibiotic resistance, streptococcus pneumoniae, pneumococcal, vaccine, children

## Abstract

After increasing from 1995 to 1999, invasive erythromycin-nonsusceptible *Streptococcus pneumoniae* rates per 100,000 decreased 53.6% in children from Baltimore, Maryland (US), from 1999 to 2001, which was partially attributed to strains related to the *mef*E-carrying England^14^-9 clone. The decline in infection rates was likely due to the pneumococcal 7-valent conjugate vaccine.

From 1995 to 1999, the prevalence of macrolide resistance among invasive pneumococci doubled to 20% in the United States (1). The rise in the 1990s was primarily due to strains with an M phenotype, a surrogate marker for the *mef*E gene (1,2). In 1999, children <5 years of age and white persons had a higher proportion of M phenotype strains causing invasive disease than did older persons and black persons (1). Most macrolide-resistant strains in the United States were also penicillin-nonsusceptible (1); modeling suggested that strains resistant to both drug classes would increase without a vaccine or other intervention (3). Since the introduction of the 7-valent pneumococcal conjugate vaccine (PCV7) in 2000, the overall incidence of macrolide-resistant infections, including serotype 14 strains, has decreased in Atlanta, Georgia (4). It is unclear whether these changes are caused by shifts in a small number of clones. Most drug-resistant infections in the United States are related to a small number of international clones (5).

## The Study

The Baltimore metropolitan area is one of the sites in the Active Bacterial Core system that conducts active, laboratory surveillance for invasive pneumococcal disease. Approximately 15 million people residing in Maryland, Georgia, California, Minnesota, Oregon, Tennessee, and Connecticut were included in the multicenter study (1). In this study, we calculated the rates of invasive erythromycin-nonsusceptible *S. pneumoniae* disease, *mef*E-associated disease, and disease due to *mef*E-carrying clones in the Baltimore metropolitan area in 1995, 1999, and 2001. These years were chosen to validate the earlier multicenter study in the Baltimore metropolitan area (1995 and 1999) and include 1 year after licensure of PCV7 (2001). PCV7 includes serotypes 4, 6B, 9V, 14, 18C, 19F, and 23F. We also assessed whether the *mef*E-carrying strains were equally distributed in all populations, in all locations, and during both respiratory and nonrespiratory seasons from 1995 to 2001. Cases of invasive pneumococcal disease with an erythromycin MIC ≥0.5 μg/mL isolated from January 1,1995, to December 31, 2001, were included. Strains with an erythromycin MIC ≥0.5 μg/mL and penicillin MIC ≥0.12 μg/mL were defined as erythromycin-nonsusceptible and penicillin-nonsusceptible *S. pneumoniae*, respectively (6). Pneumococcal serotypes were determined at the Centers for Disease Control and Prevention by the latex agglutination test and confirmed with Quellung reaction. The presence of *mef* and/or *ermB* was determined by using a duplex polymerase chain reaction (PCR) (7), and *mefA* was differentiated from *mefE* by PCR-restriction fragment length polymorphisms (8). Pulsed-field gel electrophoresis (PFGE) was performed on all strains (9). Dendrograms were created in Bionumerics (Applied Maths, Sint-Martens-Latem, Belgium) with a position tolerance of 1.5%. Strains were compared to the first 25 international clones (http://www.sph.emory.edu/PMEN/) (7). Multi-locus sequence typing was performed on 87 (24.5%) of the 349 strains (10,11), and included the spectrum of PFGE patterns. Based on PFGE patterns with ≥80% relatedness by dendrogram, and/or 5 identical alleles, strains were classified into sequence type (ST)-complexes. The following 4 ST-complexes were classified as the clones in this analysis: ST9-complex (related to England^14^-9 clone), ST81-complex (related to Spain^23F^-1 clone), ST156-complex (related to Spain^9V^-3 clone), and ST236-complex (related to the Taiwan^19F^-14 clone).

Rates were calculated by using population estimates from US Census Bureau data for the Baltimore metropolitan area for 1995, 1999, and 2001. Chi-square and Fisher exact tests were used to compare the proportion of the population with disease in 1995, 1999, and 2001 (SAS 8.2, SAS Institute, Cary, NC, USA). Cochran-Armitage trend test was used to compare the proportion of erythromycin-nonsusceptible *S. pneumoniae* strains carrying the *mef*E gene from 1995 to 2001. Age groups were defined as children <5 years of age and persons ≥5 years of age; races were defined as persons of white and black race (1); respiratory and nonrespiratory seasons were defined as November–April and May–October, respectively (12).

Most cases of invasive pneumococcal disease occurred in 3 geographic regions: 61.0% (2,976/4,885) in Baltimore City, 18.3% (895/4,885) in Baltimore County, and 9.8% (480/4,885) in Anne Arundel County. From January 1, 1995, to December 31, 2001, a total of 4,885 pneumococcal cases were detected in the Baltimore metropolitan area, of which 85.8% (4,192/4,885) were available for MIC testing. Ninety-seven percent (349/360) of the erythromycin-nonsusceptible *S. pneumoniae* isolates were available for further analysis. Of these isolates, 255 (73.1%) carried only the *mef*E gene, 61 (17.5%) carried only the *erm*B gene, 8 (2.3%) carried both the *mef*E and *erm*B genes, 6 (1.7%) carried the *mef*A gene, and 19 (5.4%) had unknown resistance mechanisms. All isolates carrying both the *erm*B and *mef*E genes were serogroup 19 strains that were related to the Taiwan^19F^-14 clone. The *mef*A-carrying strains were either serotype 6B or serotype 14. The serotype 6B strains, belonging to ST146, were detected in Baltimore City during a 3-month period in 1998; the serotype 14 strains were detected in Howard County in 3 different years.

The incidence of invasive pneumococcal disease significantly increased from 1995 to 1999 before dramatically decreasing from 1999 to 2001. From 1995 to 1999, the overall rates of erythromycin-nonsusceptible *S. pneumoniae* and *mef*E-associated disease increased, and then stabilized from 1999 to 2001 (Table). While the overall rates of erythromycin-nonsusceptible *S. pneumoniae* were stable, the proportion of pneumococcal strains with reduced susceptibility to erythromycin increased from 5.1% (26/510) in 1995 to 13.6% (77/567) in 2001 (p<0.01). Moreover, the proportion of erythromycin-nonsusceptible *S. pneumoniae* strains carrying the *mef*E gene with an erythromycin MIC ≥16 μg/mL increased from 0% (0/12) in 1995 to 12.3% (8/65) in 2001 (χ^2^ for linear trend, p = 0.02). The proportion of erythromycin-nonsusceptible *S. pneumoniae* strains carrying the *mef*E gene increased from 48.0% (12/25) in 1995 to 85.5% (65/76) in 2001 (p<0.01). Of the erythromycin-nonsusceptible *S. pneumoniae* strains, the proportion of *mef*E-carrying strains that were penicillin-nonsusceptible *S. pneumoniae* rose from 20.0% (5/25) in 1995 to 72.4% (55/76) in 2001 (p<0.01). In 3 counties, in both age groups, in both races, and during both seasons, the proportion of *mef*E-carrying strains increased from 1995 to 2001 (Figure; p values for all analyses were ≤0.02). Sixty-nine percent (182/263) of the *mef*E-carrying strains were related to 4 international clones (percent serotype): 30.0% England^14^-9 clone (100% 14); 16.0% Spain^23F^-1 clone (83.3% 23F); 14.1% Spain^9V^-3 clone (97.3% 9V); and 9.1% Taiwan^19F^-14 clone (95.8% 19F).

**Table:** Annual rates of erythromycin-nonsusceptible pneumococcocal disease*

Age group	Disease	1995		1999		2001		1995 vs. 1999		1999 vs. 2001	
		No. of cases	Rate	No. of cases	Rate	No. of cases	Rate	Change in rate (%)	p value	Change in rate (%)	p value
All	All invasive	683	28.1	762	31.1	599	23.6	10.7	0.05	-24.1	<0.01
	Erythromycin-nonsusceptible *S. pneumoniae*	26	1.4	71	3.2	77	3.2	125.3	<0.01	-0.7	1.0
	*mef*E	12	0.7	51	2.3	65	2.7	250.7	<0.01	16.7	0.4
	England^14^-9, *mef*E	5	0.3	19	0.9	23	1.0	213.6	0.01	10.8	0.8
<5 y	All invasive	135	76.7	146	89.3	63	38.1	16.4	0.2	-57.4	<0.01
	Erythromycin-nonsusceptible *S. pneumoniae*	8	6.1	23	15.6	11	7.2	153.5	0.01	-53.6	0.03
	*mef*E	3	2.3	18	12.2	9	5.9	429	<0.01	-51.5	0.07
	England^14^-9, *mef*E	1	0.8	11	7.4	4	2.6	870	<0.01	-64.7	0.05
≥5 y	All invasive	548	24.3	616	26.9	536	22.6	10.8	0.08	-16.1	<0.01
	Erythromycin-nonsusceptible *S. pneumoniae*	18	1.1	48	2.3	66	2.9	119	<0.01	25.2	0.2
	*mef*E	9	0.5	33	1.6	56	2.5	201.2	<0.01	54.6	0.04
	England^14^-9, *mef*E	4	0.2	8	0.4	19	0.8	64.3	0.4	116.3	0.06

*Rate calculations: For cases where an isolate was not available, erythromycin susceptibility, presence of *mef*E gene, and serotype were assumed to be similar to those for which an isolate was available. For each disease category, rates >2 for ≥1 year in any age group are included in the table.

**Figure:**
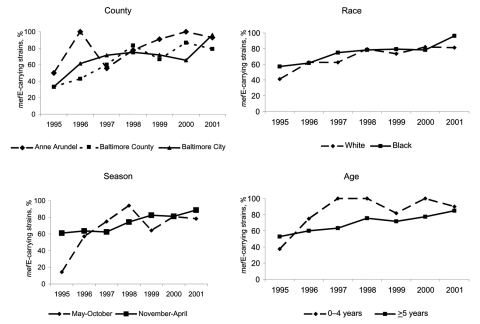
From 1995 to 2001, the proportion of erythromycin-nonsusceptible pneumococcal strains carrying the *mef*E gene increased over time by county, season, race, and age group. p<0.01 for all trends except for Anne Arundel County and May–October season (p = 0.02 for those comparisons).

Among children <5 years old, the incidence of erythromycin-nonsusceptible *S. pneumoniae* increased from 1995 to 1999 before declining by 53.6% (p = 0.03) between 1999 and 2001. Likewise, the incidence of *mef*E-associated disease rose in the 1990s before decreasing by 51.5% from 1999 to 2001 (p = 0.07). The rates of disease due to strains related to the England^14^-9 clone mirrored the age-specific trends from 1995 to 2001 (p≤0.05; Table). In contrast, in persons ≥5 years of age, the rate of erythromycin-nonsusceptible *S. pneumoniae* disease remained stable after increasing in the 1990s. From 1999 to 2001, the rate of *mef*E-associated resistance increased by 54.6% (p = 0.04; Table). Strains related to the England^14^-9 clone partially accounted for this increase.

## Conclusions

The incidence of invasive disease in the Baltimore metropolitan area increased in the 1990s before declining from 1999 to 2001 (13). Likewise, the rate of invasive erythromycin-nonsusceptible *S. pneumoniae* disease increased in the 1990s (1). From 1999 to 2001, the overall incidence of erythromycin-nonsusceptible *S. pneumoniae* disease and *mef*E-associated disease stabilized. The proportion of erythromycin-nonsusceptible *S. pneumoniae* strains carrying the *mef*E gene dramatically increased from 1995 to 2001. The increase in the proportion of erythromycin-nonsusceptible *S. pneumoniae* strains carrying the *mef*E gene over time was detected in patients residing in all 3 geographic locations, from both races, from both age groups, and during both the respiratory and nonrespiratory seasons.

Both the incidence of invasive erythromycin-nonsusceptible *S. pneumoniae* disease and *mef*E-associated disease declined by >50% from 1999 to 2001 in children <5 years of age. This decrease was partially due to serotype 14 strains related to the England^14^-9 clone. Strains related to this clone may also account for the substantial decrease in macrolide-resistant serotype 14 infections noted in Atlanta (4). In contrast to the Atlanta study, the rates of *mef*E-associated disease increased among persons ≥5 years. The differences in rates detected in Baltimore compared to Atlanta may reflect regional variation and the inclusion of 2002 in the Atlanta analysis (4). In this study, the decrease in the incidence of erythromycin-nonsusceptible *S. pneumoniae* in children may have been due to variation in antimicrobial drug use or to introduction of PCV7.

In summary, after increasing in the 1990s, the rates of invasive erythromycin-nonsusceptible *S. pneumoniae* disease stabilized overall and decreased in children from 1999 to 2001. This remarkable decline was most likely due to PCV7, although differential antimicrobial drug may have been a contributor. Unfortunately, the lack of decline in the rate of invasive erythromycin-nonsusceptible *S. pneumoniae* infections among persons ≥5 years of age, coupled with the marked increase in dual resistance and the increase in the proportion with erythromycin MICs ≥16 μg/mL from 1995 to 2001 (14), is cause for concern. Public health initiatives that focus on judicious use of antimicrobial drugs and the PCV7 vaccine (13) may be beneficial in slowing these trends (15).
